# Does new instrument for Oxford unicompartmental knee arthroplasty improve short-term clinical outcome and component alignment? A meta-analysis

**DOI:** 10.1186/s13018-020-01926-w

**Published:** 2020-09-07

**Authors:** Xiao Wei Sun, Fei Fan Lu, Kun Zou, Mao Hong, Qi Dong Zhang, Wan Shou Guo

**Affiliations:** 1 Graduate School of Peking Union Medical College and Chinese Academy of Medical Sciences, Beijing, China; 2 Department of Orthopaedic Surgery, China-Japan Friendship Hospital, No. 2 Yinghuadong Road, Chaoyang District, Beijing, 100029 China; 3 China-Japan Friendship School of Clinical Medicine, Peking University, Beijing, China; 4 Beijing University of Chinese Medicine, Beijing, China

**Keywords:** UKA, Microplasty, Clinical result, Radiological assessment

## Abstract

**Background:**

The Microplasty (MP) instrumentation designed for the Phase III Oxford mobile-bearing unicompartmental knee arthroplasty (UKA) system is considered a better option to achieve more accurate component positioning and alignment. In the present study, we focused on short-term clinical and radiological outcomes to determine whether the MP instrumentation can reduce the short-term revision rate and occurrence of outliers of metallic components.

**Methods:**

The literature in PubMed, Embase, the Cochrane Library, and Web of Science was searched up to May 2020. Studies were scrutinized by two independent authors, and the revision rate, complication spectrum, and radiological assessment with outlier rates were specifically analyzed. RevMan 5.3 was used for the statistical analysis.

**Results:**

Seven studies were included in the meta-analysis. Four studies reported both clinical and radiological outcomes, two reported only radiological outcomes, and one reported only clinical outcomes. The pooled analysis showed that the revision rate in the MP instrumentation group was 0.866 per 100 component years, while that in the control group was 1.124 (odds ratio, 0.77; *p* < 0.05). The subgroup analysis of the bearing dislocation rate showed a significantly greater reduction in the Korean population than in the populations of other countries (*p* < 0.05). The radiological assessment showed that the alignment of the femoral component was significantly improved (*p* < 0.05), while that of the tibial component was not (*p* > 0.05).

**Conclusion:**

The newly developed MP instrumentation for Oxford UKA significantly reduced the revision rate of this treatment. The positioning of the femoral component was also proven to be better by radiological assessments.

## Introduction

Various studies and systematic reviews have suggested that unicompartmental knee arthroplasty (UKA) provides a better clinical outcome but a higher revision rate than total knee arthroplasty [1–3]. Most researchers believe that the relatively poor performance of UKA may be a result of the unsatisfactory position of the metallic components [4–6]. In 2012, the Microplasty instrumentation (MP) for the Oxford unicompartmental knee system (Zimmer Biomet, Warsaw, IN, USA) was introduced to surgeons worldwide in an effort to resolve this problem [7, 8].

The MP instrumentation system, which is designed for the Phase III Oxford mobile-bearing UKA system, contains a G-clamp connecting the femoral sizing spoon and the tibial resection template to ensure the appropriate tibial resection level as well as an intramedullary rod that links to the femoral drill guide in a parallel manner to ensure the appropriate femoral component sagittal alignment. Theoretically, using this new instrument can achieve better component positioning and alignment, which may also lead to improved clinical outcomes and reduced revision rates. Although some short-term reports [9, 10] have provided supportive evidence, other studies have shown no significant differences in revision rates [11] or prosthesis positioning [12].

Because this new instrument for UKA has been used in clinical practice for less than 8 years, and possibly for even less time in Asian countries, the mid- and long-term results have not yet been analyzed. Therefore, in the present study, we focused only on short-term clinical and radiological outcomes. The purposes of this systematic review and meta-analysis were to (1) determine whether the MP instrumentation can reduce the short-term revision rate compared with the conventional instrumentation (CI) of the Phase III Oxford UKA system and (2) explore whether the MP instrumentation is superior to the CI in radiological assessment of the metallic implants and whether the MP instrumentation can reduce the occurrence of outliers in both the coronal and sagittal planes.

## Material and methods

### Literature search

We conducted this systematic review and meta-analysis according to the Preferred Reporting Items for Systematic Reviews and Meta-Analyses (PRISMA) statement with the checklist uploaded as an additional file. Therefore, ethical approval was not required.

The literature search was performed in multiple electronic databases (PubMed, Embase, the Cochrane Library, and Web of Science) up to May 2020. The search strategy was based on the following keywords: “UKA” or “UKR” or “UCR” or “unicompartmental knee replacement” or “unicompartmental knee arthroplasty” or “unicondylar replacement” or “unicondylar arthroplasty” and “Microplasty” or “MP” or “device” or “instrumentation.” We also manually searched the reference lists of relevant articles to identify studies that might have been missed in the primary search.

### Study selection

**Fig. 1:**
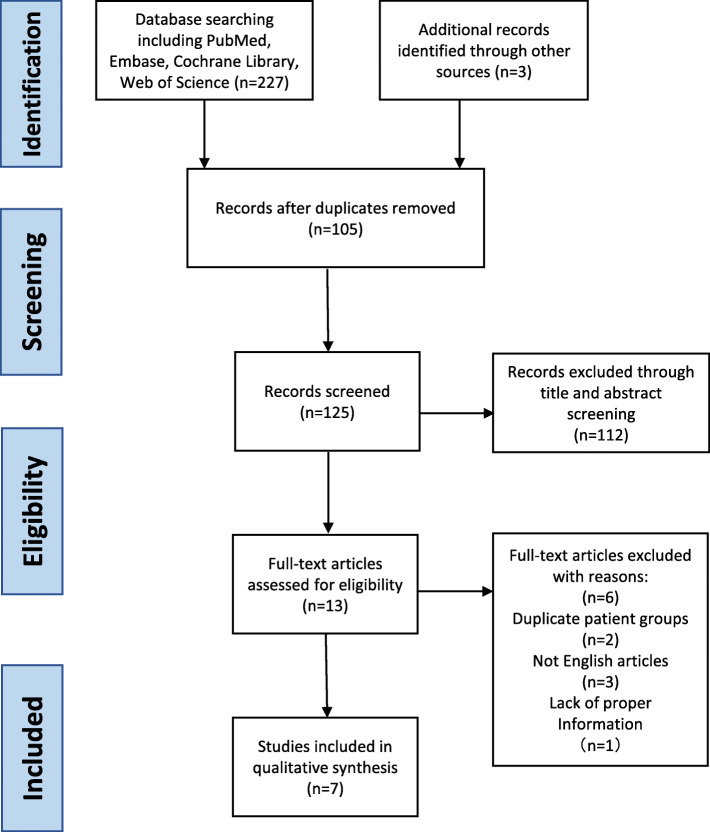
Flow diagram of literature search with inclusion and exclusion criteria

The study inclusion criteria were as follows: (1) comparable patient cohorts that underwent Phase III mobile-bearing Oxford UKA using MP instrumentation and CI; (2) either clinical outcomes or radiological assessments were reported; (3) for the clinical outcomes, detailed complications (especially early revisions) were reported; and (4) for the radiological assessments, outliers of the optimal position for both the femoral and tibial components were reported. Studies were ruled out if they met any of the following exclusion criteria: (1) written in a language other than English; (2) case series without a control group or different articles with duplicated patient groups; (3) biomechanical, cadaveric, or any type of in vitro study; and (4) studies that did not meet either inclusion criterion (3) or (4). However, because MP instrumentation has been used in clinical practice for only 6 to 8 years, very few reports met our standards. We identified only 13 eligible articles for our study [7–19]. After scrutinizing the full text, we excluded three articles not written in English [7, 16, 17], two articles by the same authors and describing the same patient group [14, 15], and one article in which the complications were not fully reported [18]. Finally, seven articles were included in the meta-analysis [8–13, 19]. The data search and selection process is shown as a flow diagram in Fig. 1.

### Data extraction

We designed a four-part electronic data form for data extraction including (1) basic information of each study, (2) clinical results including detailed complication and revision information, (3) radiological results including angles of component alignment and outlier cases, and (4) heterogeneity factors including age, sex, body mass index, and follow-up period. For studies in which data were incomplete or unclear, attempts were made to contact the authors for details. All data were extracted by two independent authors (K.Z. and M.H.), and any disagreement was checked by a senior surgeon and third author (QD.Z.) to reach a final decision.

### Quality assessment

Because only one article was a prospective cohort study with randomization and the other six were retrospective studies, we applied the Methodological Index for Non-Randomized Studies (MINORS) for quality assessment [20]. This evaluation system contains 12 items for comparative studies. If the information was not reported, the item was assigned a score of 0; if the information was reported but inadequate, the item was assigned a score of 1; and if the information was reported and adequate, the item was assigned a score of 2. Two authors independently performed the evaluation with a total score of 24 points for studies with clinical outcomes and 18 points for studies reporting only radiological assessments. Studies that scored > 75% of the total points were considered to have a low risk of bias. The intraclass correlation coefficient (ICC) was widely used to evaluate the interobserver and intraobserver reliability. Generally, the coefficiency was considered very strong if the ICC was > 0.8, moderately strong if the ICC was 0.6 to 0.8, fair if the ICC was 0.3 to 0.6, and weak if the ICC was < 0.3.

### Statistical analysis

The clinical and radiological results of the included studies were pooled for a meta-analysis when more than two reports were available. RevMan version 5.3 (The Nordic Cochrane Centre, The Cochrane Collaboration, Copenhagen, Denmark) was used to perform the meta-analysis. Dichotomous outcomes such as revision cases and radiological outliers were entered as the number of events, and comparison results are presented as odds ratios (ORs) and 95% confidence intervals (95% CIs). Because all of the studies reported different follow-up times for the MP instrumentation and CI groups, we adjusted the total samples with the time variable. This was calculated for both groups using the mean number of follow-up years multiplied by the number of knees. Therefore, the revision rate is described as revisions per 100 component years with the 95% CI [9, 21]. The level of statistical significance was set at *p* < 0.05. The Q test and chi-square test were used to show statistical heterogeneity. A fixed-effects model was applied if *I*^2^ < 50% and *p* > 0.1; otherwise, a random-effects model was used.

## Results

Seven studies were included in our meta-analysis. Four reported both clinical and radiological outcomes [8, 11–13], two reported only radiological outcomes [10, 19], and one reported only clinical outcomes [9]. The details are shown in Table 1. Application of the MINORS for quality assessment showed that all seven of these studies achieved > 75% of the total points, as shown in Table 2. Thus, the risk of bias was considered low in all studies. Six papers reported radiological assessments, and four of these six reported ICCs to evaluate the interobserver and intraobserver reliability. The details are shown in Table 3.

**Table 1:** Features of included studies

Article	Data source	Sample size		Gender (M/F)		Age (year)		BMI(kg/m^2^)		Follow-up period		Study design
		MP	CI	MP	CI	MP	CI	MP	CI	MP	CI	
Mohammad et al. [9]	NJR	7953	7953	4341/3612	4330/3623	64.5 ± 9.4	64.6 ± 9.5	30.6 ± 5.1	30.1 ± 4.9	2.3 ± 1.3	3.3 ± 1.8	Case control
Malhotra et al. [13]	India	100	50	28/72	9/41	58.3 ± 8.2	59.8 ± 8.4	28.7 ± 3.1	29.3 ± 2.8	1.5 ± 0.4	3.8 ± 1.4	Case control
Jang et al. [12]	Korea	77	77	10/67	3/74	65.8 ± 7.9	66.1 ± 7.6	25.6 ± 3.0	25.6 ± 2.8	1.8	6.2	Case control
Tu et al. [11]	China	56	52	21/35	19/33	67.1	66.8	30.1	29.8	2.1		Prospective randomized
Koh et al. [8]	Korea	41	41	6/35	8/33	60.3 ± 5.9	59.6 ± 8.1	26.2 ± 3.2	25.8 ± 3.9	2.8		Case control
Walker et al. [10]	Germany	100	100	54/46	45/55	63.1	63.3	30.4	30.3	NA		Case control
Hurst et al. [19]	NJR	186	219	84/87	83/105	64.1	63.0	32.6	31.9	NA		Retrospective cohort

**Table 2:** Methodological items for non-randomized studies (MINORS)

	Clearly stated study aim	Inclusion of consecutive patients	Prospective collection of data	Appropriate endpoints	Unbiased assessment of endpoint	Appropriate follow-up period	Loss to follow-up less than 5%	Prospective calculation of study size	Adequate control group	Contemporary groups	Baseline equivalence of groups	Adequate statistical analyses	Total score
Mohammad et al. [9]	2	2	2	2	2	2	2	2	2	1	2	2	23/24
Malhotra et al. [13]	2	2	2	2	1	1	2	0	2	1	2	2	19/24
Jang et al. [12]	2	2	2	2	2	1	2	0	2	1	2	2	20/24
Tu et al. [11]	2	2	2	2	2	1	2	0	2	1	2	2	22/24
Koh et al. [8]	2	2	2	2	1	1	2	0	2	1	2	2	19/24
Walker* et al. [12]	2	2	2	NA	NA	NA	NA	0	2	1	2	2	13/16
Hurst* et al. [19]	2	2	2	NA	NA	NA	NA	0	2	1	2	2	13/16

*Walker’s and Hurst’s articles reported only radiological assessments without follow-up, so that the full score should be 16 points

**Table 3:** Intraclass correlation coefficient of radiological assessment

	Intraclass correlation coefficient (ICC)	
	Intra-observer	Inter-observer
Malhotra et al. [13]	NA	NA
Jang et al. [12]	0.936-0.997	0.962-0.998
Tu et al. [11]	NA	0.75-0.85
Koh et al. [8]	0.84–0.99	0.81–0.99
Walker et al. [12]	0.89-0.97	NA
Hurst et al. [19]	NA	NA

### Clinical results

**Fig. 2:**
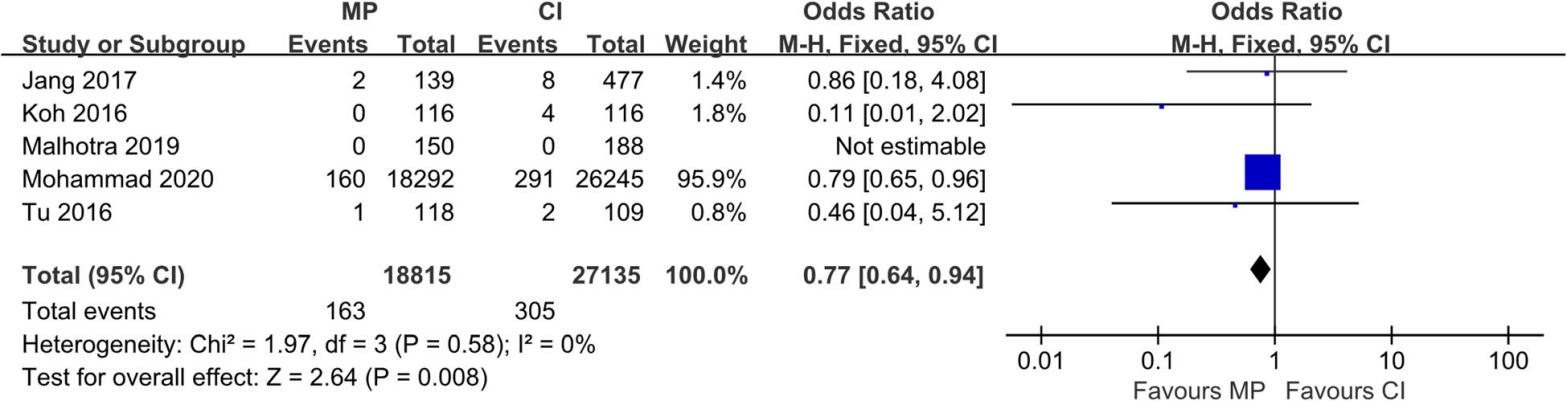
Forest plot of included studies comparing revision rate

**Fig. 3:**
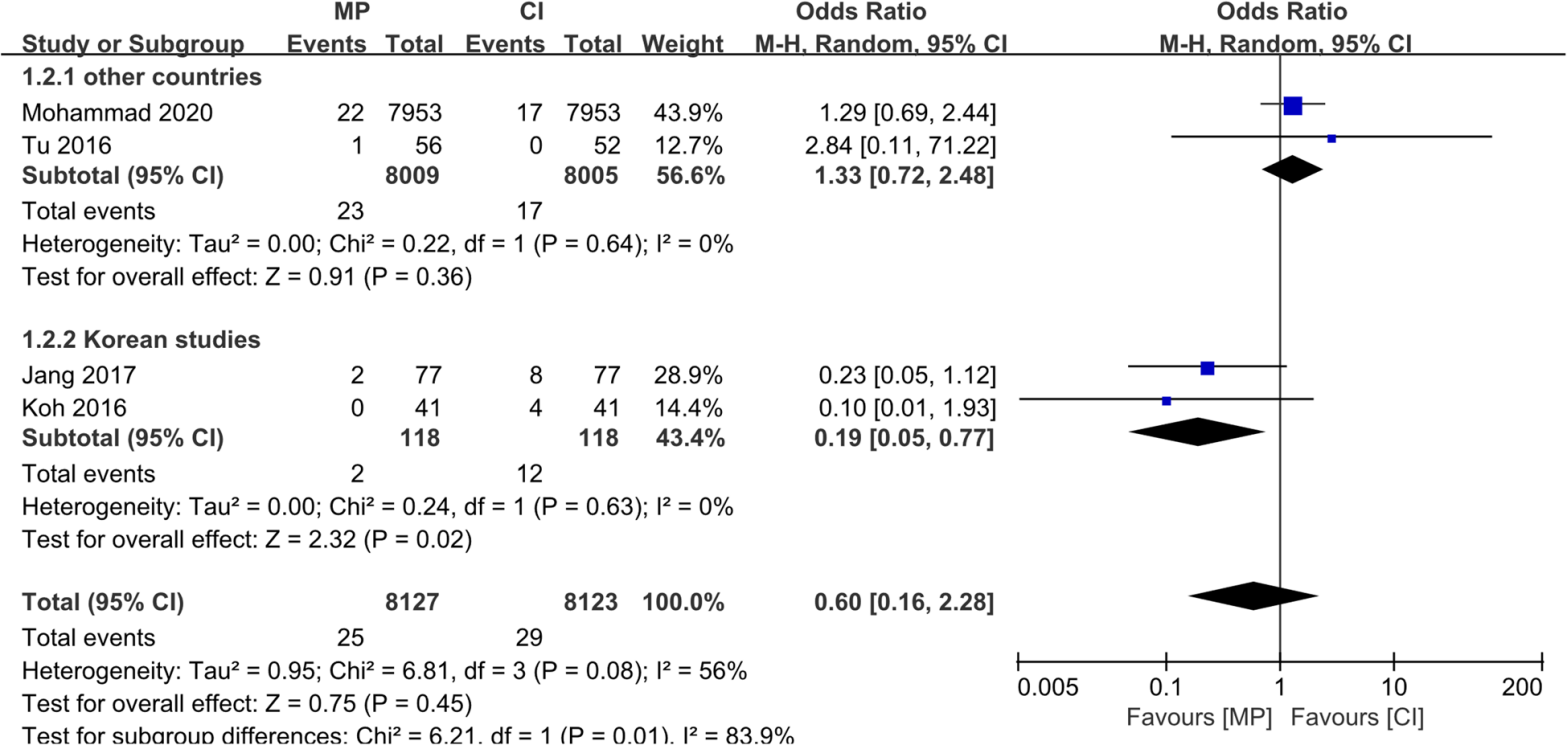
Forest plot of subgroup analysis of Korean and other studies comparing bearing dislocation rate

The revision rate of UKA was significantly lower using MP instrumentation than CI in most of the included articles. Because the new instrumentation had been in use for only a short period, a small number of cases were included in most studies. However, one case–control study with a large sample size from the National Joint Registry (NJR) investigated > 15,000 patients who underwent UKA [9]. The pooled analysis showed that the revision rate in the MP instrumentation group was 0.866 per 100 component years, while that in the CI group was 1.124 (OR, 0.77; 95% CI, 0.64–0.94; *p* < 0.05). The details are shown in Fig. 2. Additionally, dislocation of the mobile bearing was considered the only cause leading to revision in the Korean reports [8, 12], which was very different from the NJR report [9]. This difference suggested that the leading causes of revisions in both the MP instrumentation and CI groups were progression of osteoarthritis and aseptic loosening of the implants. Thus, we performed a subgroup analysis focused only on bearing dislocation between the reports from Korea and the reports from other countries. As shown in Fig. 3, the OR in the Korean subgroup was 0.19 (95% CI, 0.05–0.77; *p* < 0.05), which indicated that the MP instrumentation could significantly reduce the bearing dislocation rate in Korean patients. Additionally, the intra-subgroup difference was statistically significant (*I*^2^ = 83.9%, *p* < 0.05), which confirmed the difference between reports from Korea and reports from other countries.

### Radiological results

**Fig. 4:**
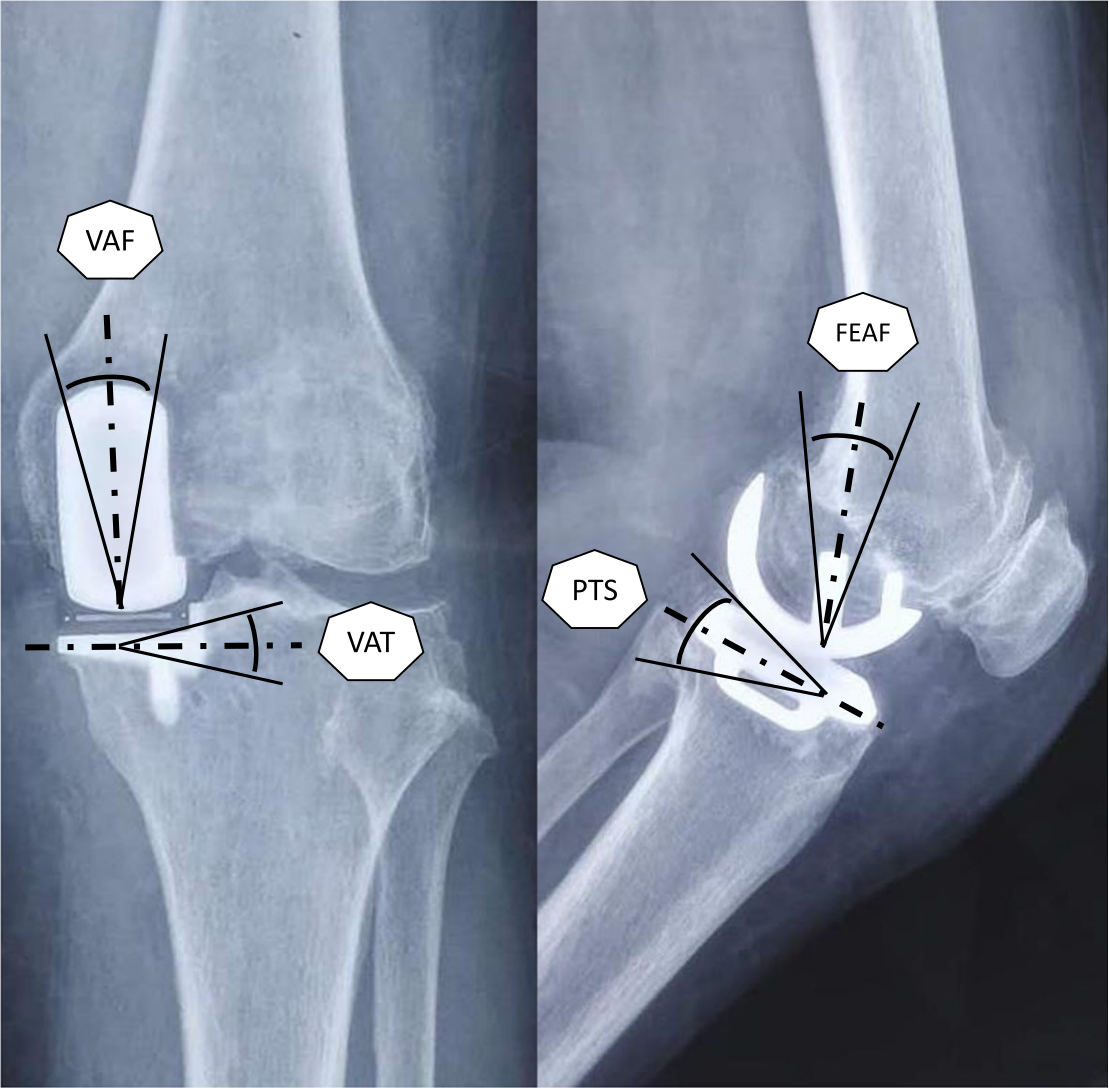
Radiological parameter measurements including four angles

**Fig. 5:**
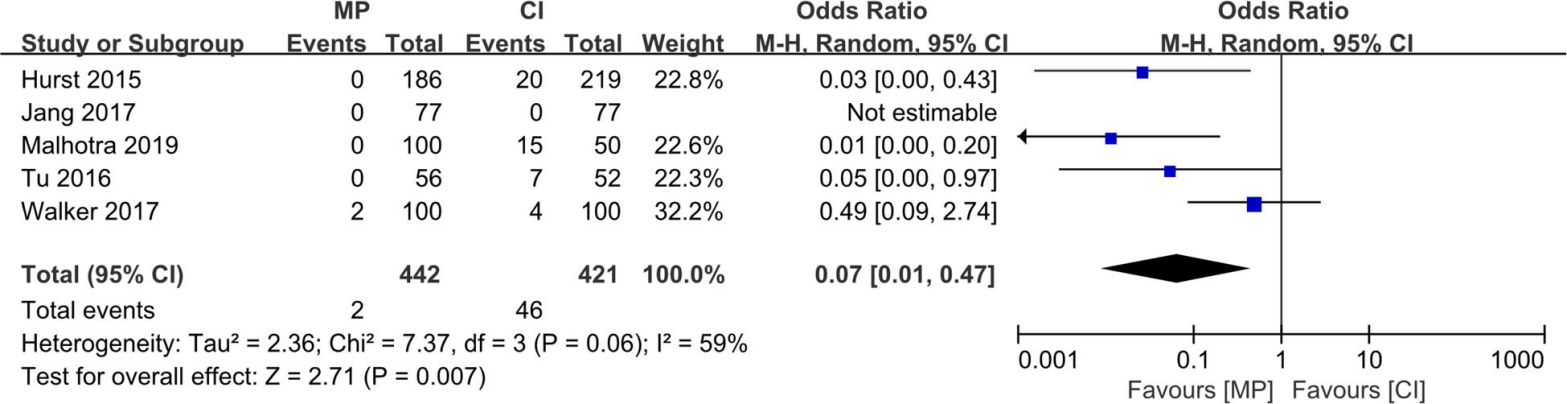
Forest plot of included studies comparing outliers of VAF

**Fig. 6:**
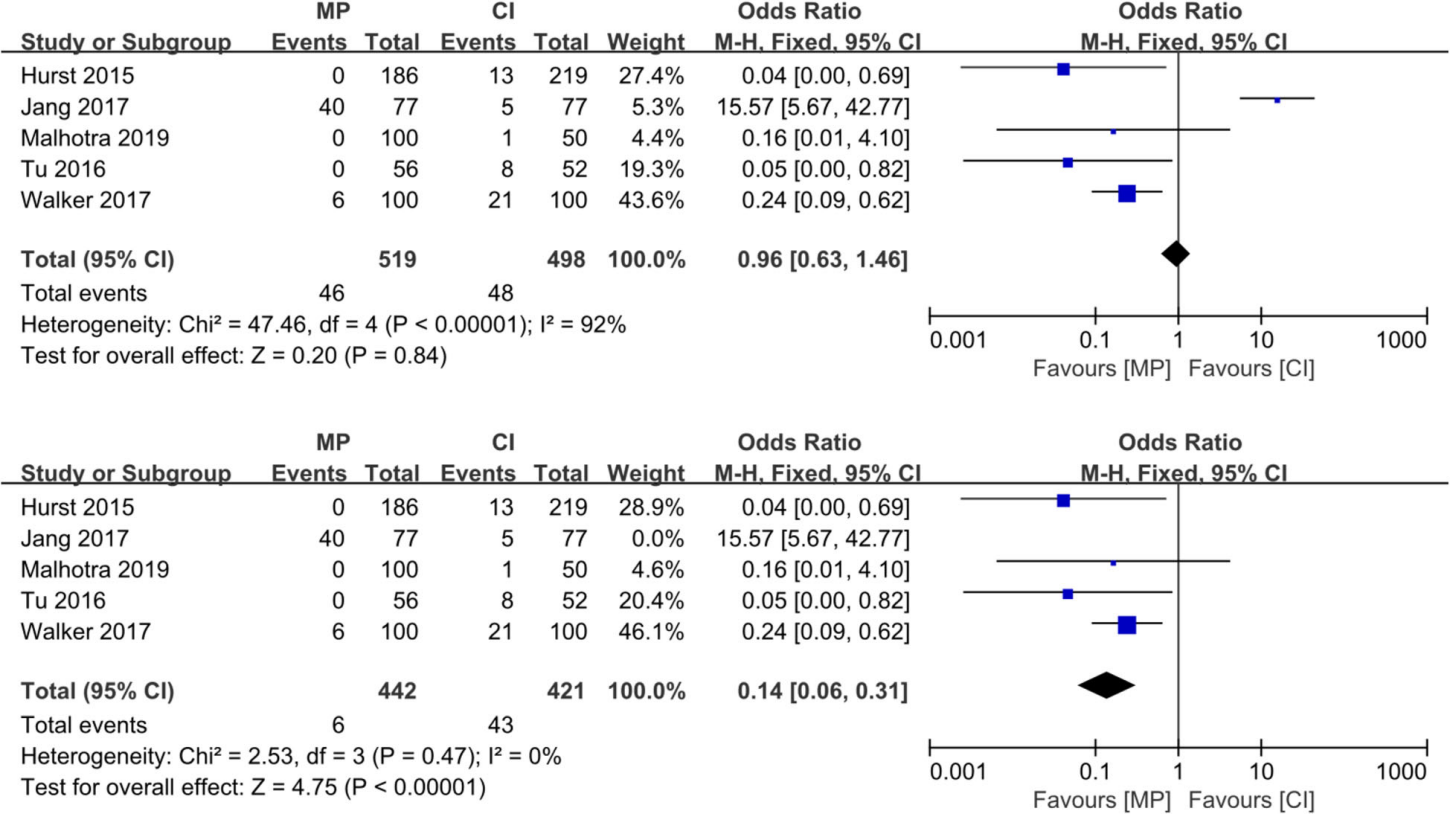
Forest plot of included studies comparing outliers of FEAF. (Above) The heterogeneity of the included studies was high. (Below) When the study by Jang et al. was excluded, the heterogeneity significantly decreased (*I*^2^ = 0%)

**Fig. 7:**
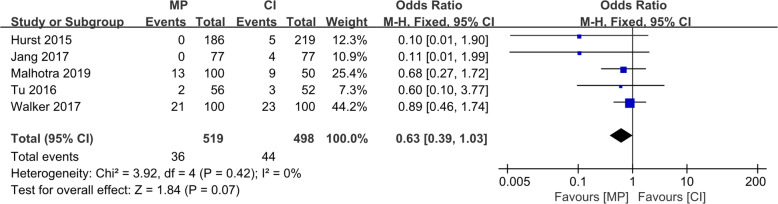
Forest plot of included studies comparing outliers of VAT

**Fig. 8:**
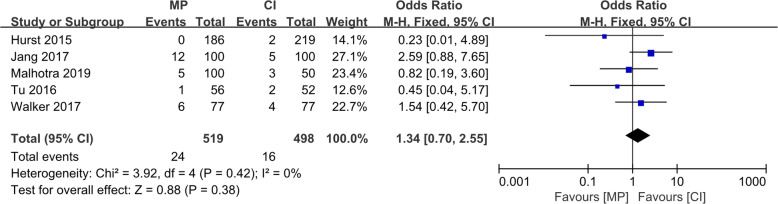
Forest plot of included studies comparing outliers of PTS

Although the radiological results such as the implant positions were estimated differently in various reports, all six studies reported four essential factors: (1) the varus/valgus angle of the femoral component (VAF), representing its alignment in the coronal plane; (2) the flexion/extension angle of the femoral component (FEAF), representing its alignment in the sagittal plane; (3) the varus/valgus angle of the tibial component (VAT), representing its alignment in the coronal plane; and (4) the posterior tibial slope (PTS), representing the tibial component alignment in the sagittal plane. The definitions of these angles are shown in Fig. 4. The conception of outliers was introduced to evaluate the accuracy of the implant position because the optimal alignment has been described as a range of angles [10, 19]. We conducted a meta-analysis of the five reports with outlier information of the four essential angles. For the VAF, the OR was 0.07 (95% CI, 0.01–0.47; *p* < 0.05), indicating that the MP instrumentation could significantly reduce the outliers of the femoral implant in the coronal plane as shown in Fig. 5. For the FEAF, the OR was 0.96 (95% CI, 0.63–1.46; *p* > 0.05) with high heterogeneity (*I*^2^ = 92%). However, after we removed the study by Jang et al. [12], the OR sharply decreased to 0.14 (95% CI, 0.06–0.31; *p* < 0.05) with an *I*^2^ value of 0%. This result also suggests that the MP instrumentation could significantly reduce the outliers of the femoral implant in the sagittal plane. The details are shown in Fig. 6. The VAT and PTS were also pooled for analysis; however, the results showed no significant differences in outliers between the MP instrumentation and CI groups. These details are shown in Figs. 7 and 8.

## Discussion

The MP instrumentation was designed by the Oxford group and was initially used in British hospitals. Beginning in 2012, it was introduced worldwide. Thus, few studies have reported the clinical outcomes of MP instrumentation before 2020, and these few studies had sample sizes of no more than 200 cases. Using the NJR data, Mohammad et al. [9] was able to compare MP and non-MP UKA in a large sample. In a well-designed case–control study of > 15,000 cases, the authors found that the 5-year survival rate after MP UKA was 96.7%, which was significantly better than that in the control group (94.5%). This is very solid evidence that using MP instrumentation can achieve a better outcome of UKA. With respect to the huge sample size, this report inevitably had substantial weight on the revision rate of our meta-analysis, which provided a very similar result.

These improvements in clinical outcomes were considered by most researchers to be a result of better implant positions and better alignment. As shown by the results of the radiological assessments in the present meta-analysis, MP instrumentation can achieve a better position of the femoral component with respect to both the VAF and FEAF. Hurst et al. [19] suggested that this was mainly because of the newly designed linking device that connected the intramedullary rod and femoral drill guide. Because this linking can limit the drill guide in both the coronal and sagittal planes, the femoral milling was more accurate than using CI, resulting in more accurate positioning of the femoral component. The findings reported by Koh et al. [8] and Tu et al. [11] are also in agreement with this theory. Furthermore, Tu et al. [11] suggested that the intramedullary rod of MP instrumentation was both longer and thicker than that of CI; this can limit the micromotion of the intramedullary rod itself, leading to a more repeatable surgical procedure and more reliable alignment. However, during the pooled analysis of the FEAF, we removed the study by Jang et al. [12] because of high heterogeneity. Many more outliers in the sagittal axis of the femoral component (FEAF) were observed in the MP instrumentation group in this report, which was opposite of other studies. The researchers considered that one possible explanation was the individual differences in the femoral anatomy, especially in terms of the increased anterior bowing in the Asian population. In contrast, Walker et al. [10] suggested that the insertion point of the intramedullary rod can remarkably affect the sagittal axis of the femoral component, which may be the main cause of the outliers of sagittal alignment. This theory was also proven in a cadaver study by Kort et al. [22], who suggested that an accurate insertion point as well as a relatively longer intramedullary rod could improve the alignment of the femoral component of UKA.

The major improvement of the MP instrumentation is the intramedullary rod with the linking device to the femoral mill, which does not influence the position of the tibial component. Our meta-analysis also showed no significant difference in either the VAT or PTS between the MP instrumentation and CI groups. Theoretically, the G-clamp can improve the accuracy of the tibial implant in the coronal plane with fewer outliers of the VAT. A possible explanation is that the resection of the tibial plateau is mainly determined by the extramedullary measurement of the tibia, which overtakes the smaller influence of the G-clamp.

Another factor that should be noted is the various causes of revisions. The complications leading to revision were different between reports from Korea and reports from other countries. In the studies by Jang et al. [12] and Koh et al. [8], all 14 revisions were performed because of mobile-bearing dislocations, which were considered the most common cause of early failure of UKA. The introduction of MP instrumentation might have significantly reduced the dislocation rate; however, other reports have suggested that using MP instrumentation can hardly achieve a reduction in the dislocation rate. Our subgroup meta-analysis showed that the differences between subgroups were statistically significant. The high dislocation rate might have been due to the frequently performed deep knee flexion as part of the patients’ traditional lifestyle as well as religious practices involving the kneeling-sitting or cross-leg sitting positions [21]. Because the new instrument would not change any of these lifestyle factors, there must be another explanation.

Pandit et al. [23] demonstrated that the bearing size could be a risk factor for poor long-term survival after UKA. They showed that the 15-year survival rate was 75% in a group of patients with a bearing size of > 5 mm. However, the 3- and 4-mm bearing group achieved a survival rate of 94%. These results implied that the need for a thicker bearing may be due to deep tibial resection, which may lead to multiple complications, especially mobile-bearing dislocation. The newly designed MP instrumentation can link the femoral sizing spoon to the tibial resection plate with a G-clamp, which partly restricts the coronal plane of tibial resection and reduces the depth. Several reports have confirmed that MP instrumentation can significantly reduce the bearing size or depth of tibial resection by radiographic measurement [8, 10, 19]. However, various methods were used and could not be pooled together for analysis. Nevertheless, all three studies from Korea showed that MP instrumentation could significantly reduce the bearing dislocation rate [8, 12, 18].

This review had a few limitations. First, it included only one randomized prospective study; most of the others were case–control studies. Second, UKA has very different complication spectrums between Western and Asian patients [21]. In our review, however, the NJR report had substantial weight on the assessment of the clinical outcomes, while the Asian reports contributed only minor effects; this may have led to a selective bias. Third, the radiographic measurement parameters varied among the studies. Only four angles were common to all studies, but they still showed minimal differences in the definition of outliers.

## Conclusion

The newly developed MP instrumentation for Oxford UKA significantly reduced the revision rate of this treatment. Additionally, this instrument significantly reduced the bearing dislocation rates in Korean patients. The positioning of the femoral component was also proven to be better by radiological assessments.

## Supplementary information

**Additional file 1.** PRISMA Checklist

**Additional file 2.**

## Data Availability

The datasets generated and/or analyzed during the current study are available from the corresponding author on reasonable request.

## Abbreviations

MP: Microplasty
UKA: Unicompartmental knee arthroplasty
CI: Conventional instrumentation
PRISMA: Preferred Reporting Items for Systematic Reviews and Meta-Analyses
MINORS: Methodological Index for Non-Randomized Studies
ICC: Intraclass correlation coefficient
OR: Odds ratio
95% CI: 95% confidence interval
NJR: National Joint Registry
VAF: Varus/valgus angle of femoral component
FEAF: Flexion/extension angle of femoral component
VAT: Varus/valgus angle of tibial component
PTS: Posterior tibial slope
